# Dentate Gyrus Volume Mediates the Effect of Fornix Microstructure on Memory Formation in Older Adults

**DOI:** 10.3389/fnagi.2020.00079

**Published:** 2020-03-20

**Authors:** Dayana Hayek, Friederike Thams, Agnes Flöel, Daria Antonenko

**Affiliations:** ^1^Department of Neurology, NeuroCure Clinical Research Center, Berlin Institute of Health, Corporate Member of Freie Universität Berlin, Charité – Universitätsmedizin Berlin, Humboldt-Universität Berlin, Berlin, Germany; ^2^Department of Neurology, Universitätsmedizin Greifswald, Greifswald, Germany; ^3^German Centre for Neurodegenerative Diseases (DZNE) Standort Greifswald, Greifswald, Germany

**Keywords:** aging, brain plasticity, white matter integrity, hippocampal subfields, tractography

## Abstract

Age-related deterioration in white and gray matter is linked to cognitive deficits. Reduced microstructure of the fornix, the major efferent pathway of the hippocampus, and volume of the dentate gyrus (DG), may cause age-associated memory decline. However, the linkage between these anatomical determinants and memory retrieval in healthy aging are poorly understood. In 30 older adults, we acquired diffusion tensor and T1-weighted images for individual deterministic tractography and volume estimation. A memory task, administered outside of the scanner to assess retrieval of learned associations, required discrimination of previously acquired picture-word pairs. The results showed that fornix fractional anisotropy (FA) and left DG volumes were related to successful retrieval. These brain-behavior associations were observed for correct rejections, but not hits, indicating specificity of memory network functioning for detecting false associations. Mediation analyses showed that left DG volume mediated the effect of fornix FA on memory (48%), but not vice versa. These findings suggest that reduced microstructure induces volume loss and thus negatively affects retrieval of learned associations, complementing evidence of a pivotal role of the fornix in healthy aging. Our study offers a neurobehavioral model to explain variability in memory retrieval in older adults, an important prerequisite for the development of interventions to counteract cognitive decline.

## Introduction

Age-related deterioration of white and gray matter in the human brain contributes to cognitive impairment in the course of aging (Grady, 2012; Marstaller et al., 2015). Microstructure in white matter tracts as assessed by diffusion tensor imaging (DTI) and gray matter volume estimated from high-resolution structural magnetic resonance images predict behavioral task performance in healthy older adults (Antonenko et al., 2012; Sasson et al., 2013). Episodic memory, as one of the most vulnerable cognitive domains in aging (Nyberg et al., 2012), is mediated by hippocampal networks, including the hippocampus itself and the fornix as its major efferent white matter pathway (Metzler-Baddeley et al., 2011; Lovblad et al., 2014; Antonenko et al., 2016b; Gorbach et al., 2017; Anblagan et al., 2018).

Reduced fornix white matter microstructure has been observed in the course of healthy, but also pathological aging processes (Metzler-Baddeley et al., 2012; Pelletier et al., 2013; Zhuang et al., 2013). Deteriorated fornix pathways were suggested to be one of the earliest abnormalities in older individuals with mild cognitive impairment (MCI) who progress to Alzheimer’s dementia (AD) (Douaud et al., 2013; Kantarci, 2014). Inter-individual variability in fornix macro- and microstructure has been associated with older adults’ variability in performance on various episodic memory tasks that require verbal or visual recall or recognition of items and associations (Metzler-Baddeley et al., 2011; Lockhart et al., 2012; Douet and Chang, 2014; Henson et al., 2016; Gazes et al., 2018). Likewise, the hippocampus is susceptible to healthy and pathological aging, leading to altered episodic memory function (Frisoni et al., 2008; Walhovd et al., 2011; den Heijer et al., 2012). Whether or not volumetric atrophy of the whole hippocampus contributes to age-related memory decline is unclear (Head et al., 2005; Fjell et al., 2010; Pereira et al., 2014). This may be due to differential vulnerability of its subfields to aging (Small et al., 2002, 2011). Within the hippocampal formation, advanced age has been associated mainly with reduced volumes in its subfields cornu ammonis 1–4 and dentate gyrus (DG) (Mueller and Weiner, 2009; Pereira et al., 2014). The DG was observed to have a stronger age-associated cerebral blood volume decrease compared to other hippocampal subfields (Brickman et al., 2014). Due to its particular vulnerability to age-related processes (Small et al., 2004; Wilson et al., 2006) and activity during memory discrimination tasks, changes in DG has been suggested to drive age-related cognitive decline (Yassa et al., 2011a, b; Doxey and Kirwan, 2015).

Of particular importance in the context of healthy aging and AD is the question of mutual dependency of gray and white matter damage, i.e., their directional relationship and interaction to predict cognitive decline (cf. Bartzokis, 2011; Dansokho and Heneka, 2018; Metzler-Baddeley et al., 2019). Within structural hippocampal networks, correlational studies have found positive associations between hippocampal atrophy and loss of fornix connections in older adults, with findings pointing toward a pivotal role of the latter in older age (Fletcher et al., 2013; Pelletier et al., 2013; Zhuang et al., 2013; Gazes et al., 2018). Using mediation analyses, a recent study applied individual white matter fiber tractography and hippocampal segmentation to investigate the linkage between increased age, reduced fornix microstructure and hippocampal atrophy in a directional approach (Metzler-Baddeley et al., 2019). The results revealed that white matter changes predicted gray matter deterioration, but not vice versa, concordant with the idea of age-related myelin damage causing abnormal intracellular metabolism and neuronal death (Bartzokis, 2004; Metzler-Baddeley et al., 2019). Even with evidence from previous research showing a structural connectivity between fornix and subiculum (Saunders and Aggleton, 2007), animal studies have observed a unique functional connection between the fornix and the DG subfield (Gondard et al., 2015; Hao et al., 2015). The authors showed that deep brain stimulation of the fornix activated the DG by modulating the expression of neurotrophic factors and markers of synaptic plasticity known to be crucial for memory processing. These studies could indicate that disconnection in forniceal white matter pathways might induce functional and/or structural changes in hippocampus, and more specifically in its DG subfield, which in turn may affect age-related decline in memory function. The interactive effect of both structures on the ability to form novel memories in older adults has not been elucidated yet. In the present study, we aimed to investigate this linkage between structural hippocampal networks and the retrieval of episodic memory in healthy older adults. We administered a task that required learning of new picture-word associations and subsequent discrimination of correct and incorrect pairings during retrieval in order to assess hippocampus-dependent memory performance (Antonenko et al., 2016a, 2019). Individual forniceal pathways were reconstructed on diffusion-weighted images using deterministic tractography based on the constrained spherical deconvolution (CSD) technique. Based on previous findings suggesting the involvement of left hippocampus and its subfields in similar or the same verbal memory tasks (Breitenstein et al., 2005; Pereira et al., 2014; Witt et al., 2014; Doxey and Kirwan, 2015; Powell et al., 2016; De Shetler and Rissman, 2017; Antonenko et al., 2019), individual volumes of the left DG were estimated on T1 images using automated subcortical segmentation (Iglesias et al., 2015). We aimed to explore correlational relationships between memory retrieval performance in different response categories reflecting the detection of correct and incorrect associations, fractional anisotropy (FA) in the fornix and volume in the left DG. Subsequent mediation models were conducted to evaluate the linkage between structural properties and memory performance.

## Materials and Methods

### Participants and Study Design

Thirty healthy older subjects between the age of 50 and 79 years were recruited in this study (14 f; mean/SD age: 62/6 years). They were all right-handed, German native speakers, with no history of neurological diseases. Neuropsychological testing was performed for all participants to assure normal cognitive functioning within age- and education-related norms (CERAD-Plus)^1^ (Table 1). The study was approved by the Ethics Committee of the Charité University Medicine and conducted in accordance with the Helsinki Declaration. Written informed consent was obtained from all participants prior to participation.

**TABLE 1:** Characteristics of participants.

	**Mean**	**SD**
Age, years	62	6
Education, years	15	2
LQ^a^	94.3	9.3
GDS^b^	1.3	1.4
Digit Span (max. 14)		
Forward	7.6	2.4
Backward	6.1	1.9
Vocabulary test (max. 37)^c^	33.2	2.1
Semantic fluency, *N* (in 60s)	25.3	5.6
Boston Naming Test, *N* (max.15)	14.7	0.5
Mini-Mental State (max. 30)	29.4	0.9
Word list learning, *N*		
Total (max. 30)	23.3	3.0
Trial 1 (max. 10)	6.2	1.5
Trial 2 (max. 10)	8.1	1.3
Trial 3 (max. 10)	9.1	0.9
Word list retrieval, *N* (max. 10)	8.3	1.2
Word list intrusions, *N*	1.0	2.0
Figure copying, *N* (max. 11)	11	0.0
Figure retrieval, *N* (max. 11)	10.7	0.8
Phonemic fluency, *N* (in 60s)	16.1	4.1
Trail making test, *s*		
Part A	38.9	10.7
Part B	79.0	20.2

*^*a*^LQ, laterality quotient (Oldfield, 1971). ^*b*^GDS, Geriatric Depression Scale (Yesavage et al., 1982). ^*c*^Lehrl (2005).*

### Episodic Memory Task

The task was adapted from previous studies (Breitenstein and Knecht, 2002; Breitenstein et al., 2005; Floel et al., 2008; Antonenko et al., 2016a, 2019), and programmed using the software Presentation (Neurobehavioral Systems)^2^. The paradigm consists of the presentation of 30 picture-pseudoword pairs. For each participant, a set of 30 pseudowords and 30 pictures of daily life were randomly matched to generate 30 “correct” picture-pseudoword combinations (e.g., elephant = “pari”), creating a “vocabulary” list which had to be learned over the course of five blocks and retrieved in a “transfer” block (where pictures were replaced by corresponding German words). “Incorrect” pairs (e.g., elephant = “ralm”) were created by combining a picture with other pseudowords from the set. The task was originally created to resemble natural language learning, so the underlying learning principle involves the detection of higher co-occurrences of “correct” compared to “incorrect” pairs (Breitenstein and Knecht, 2002).

During the learning phase, 600 trials were presented, divided into five blocks (120 trials per block). “Correct” pairings were presented ten times in total (i.e., twice in each block, totaling up to 60 “correct” pairs per block). Each of the 30 pictures was also presented ten times with varying “incorrect” pseudowords (i.e., each picture was paired with two different “incorrect” pseudowords per block, totaling up to 60 “incorrect” pairs per block). Each “incorrect” pairing was presented only once over the course of the whole task. Trial presentation order was randomized. Participants were instructed to answer as quickly as possible by button press if the pairing was “correct” or not, being however not informed about the underlying frequency principle. No feedback was given during the task. As the learning principle of the task is based on the detection of higher co-occurrences of (arbitrarily) “correct” compared to “incorrect” couplings (ratio 10:1) over the course of the five learning blocks, participants had to guess in the beginning of the task. In each trial of the learning phase, the picture was presented 200 ms after the onset of an auditory spoken pseudoword (normalized at the same loudness and length of 600 ms, delivered over headphones) and remained on the screen for 1500 ms. Performance accuracy increased from first to last learning block, indicating that participants were able to learn the pairings (repeated-measures ANOVA showed a significant main effect of learning blocks on performance accuracy [*F*(2.5, 71.0) = 74.43, *p* < 0.001, partial eta squared = 0.72), see Supplementary Figure S1 for individual learning curves).

During the retrieval block, corresponding spoken German words were presented instead of pictures together with the pseudowords. Stimulus count, underlying frequency principle (i.e., 60 “correct” and 60 “incorrect” pairings; the latter containing again different pseudowords than in the learning phase) and trial timings were identical to those in a learning block. Total duration of the task was 35 min. The task generated four response categories: hits (i.e., classifying a “correct” pair as “correct”), correct rejections (i.e., classifying an “incorrect” pair as “incorrect”), false alarms (i.e., classifying an “incorrect” pair as “correct”), and misses (i.e., classifying a “correct” pair as “incorrect”) (Lynn and Barrett, 2014). Distribution of performance in response categories was illustrated in Supplementary Figure S2. Percentage of total correct responses and percentage of correct responses in different response categories were examined in the retrieval phase.

### MRI Acquisition

MRI was performed using 3T Siemens Trio MR-System using 12-channel head coil at the Berlin Center for Advanced Neuroimaging. First, a 3D structural high-resolution T1-weighted magnetization prepared rapid gradient echo image was acquired with the subsequent characteristics; TR = 1900 ms, TE = 2.52 ms, 192 sagittal slices, voxel size = 1.0 mm^3^ × 1.0 mm^3^ × 1.0 mm^3^, flip angle = 9^o^. Second, a diffusion-weighted spin-echo echo-planar imaging image was acquired with the subsequent characteristics; TR = 7500 ms, TE = 86 ms, 61 axial slices, voxel size = 2.3 mm^3^ × 2.3 mm^3^ × 2.3 mm^3^; 64 directions, *b*-value of 1000 s/mm^2^, 1 b0.

### MRI Data Analysis

#### Tractography and Tract Variables

**FIGURE 1:**
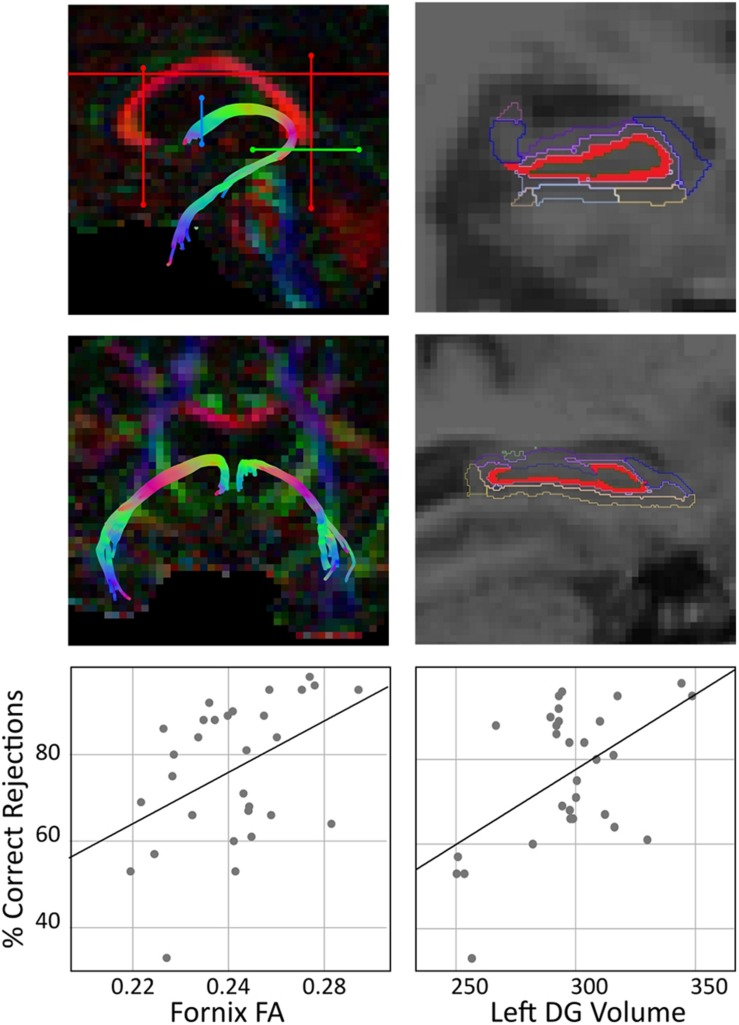
Tractography analysis, hippocampal subfields representation, and their correlation with memory performance. The left panel shows a sagittal and a coronal section of the fornix, and the tactography using ROI waypoints (Leemans et al., 2009) (“SEED” ROI is shown in blue, “AND” ROI is shown in green, and “NOT” ROIs are shown in red) for the fornix in the native space of one participant. The scatter plot shows a positive correlation of fornix FA and percentage of correct rejections, corrected for age (partial *r* = 0.403, *p* = 0.030). The right panel shows hippocampal subfields segmentation performed using FreeSurfer v6.0 algorithm. This segmentation resulted in 12 subfields; DG is shown in red and all remaining subfields are shown in colored outline. The scatter plot shows a positive correlation of left DG volume (in mm^3^) and% correct rejections, adjusted for age (partial *r* = 0.501, *p* = 0.006). Note that for both correlations, sensitivity analysis without the low performing participant was conducted and results showed similar partial correlation coefficients (correlation of fornix FA and% correct rejections: *r* = 0.416, *p* = 0.028; correlation of left DG volume and % correct rejections: *r* = 0.449, *p* = 0.017).

Tractography was performed using ExploreDTI (Leemans et al., 2009). Diffusion MRI images were corrected for eddy currents and distortions caused by head motion. Metzler-Baddeley et al. (2011) previously described that diffusion tensor model tractography (Basser et al., 2000) was not found to be the most appropriate technique to reconstruct the fornix because it is positioned next to other white matter tracts. Thus, we used deterministic tracking based on CSD which was shown to resolve crossing fibers problem (Tournier et al., 2004, 2007, 2008). At each voxel, CSD extracts peaks in the fiber orientation density function (fODF). The fODF estimates the proportion of fibers in a voxel pointing in each direction. The diffusion orientation was estimated at each seed point and then moved further in 0.5 mm steps along the direction that traversed the smallest angle of the trajectory. A pathway was depicted until either a change in direction with an angle >60^o^ or a drop of FA value below 0.2 occurred (Carballedo et al., 2012; Antonenko et al., 2016b). Whole brain tractography was performed using every voxel as seed point. In order to extract three-dimensional reconstructions of the fornix and the uncinate fasciculus, multiple way-point regions of interest (ROIs) masks were implemented. This technique uses Booleian logical operations to delineate ROIs specific masks, for instance, one can choose to reconstruct a tract that passes through ROI-1 but NOT ROI-2. Following training, all ROIs were manually drawn in native space using color-coded fiber orientation maps for individual subjects. This was performed by a single operator blinded to age, neuropsychological data and memory performance, using landmark techniques that have been defined in previously published studies and that have been shown to be highly reproducible, valid and reliable (Catani et al., 2002; Metzler-Baddeley et al., 2011; Carballedo et al., 2012), see Figure 1. The main outcome measure was mean FA. FA is one DTI variable that measures the degree of directionality of the diffusion in a specific white matter structure, and it is calculated from the diffusion tensor eigenvalues that give the directions of the diffusion (Basser and Pierpaoli, 1996). For white matter pathways, FA is the most widely used measure to examine microstructure and integrity (Alexander et al., 2007; Zatorre et al., 2012).

In order to delineate the individual tracts, first, medial level of a coronal section where the anterior pillars enter into the body of the fornix was located. “SEED” point ROI was drawn around the body of the fornix bundle. Second, at the inferior part of the splenium of the corpus callosum, “AND” ROI around the crus fornici of each hemisphere was drawn. Finally, to eliminate unwanted tracts, three “NOT” ROIs were drawn; one is rostral to the fornix pillars; one is caudal to the crus fornici, and a third one on an axial slice through the upper pons and the corpus callosum (for individual tracts, see Supplementary Figure S3). The same procedure was used to extract FA values from the uncinate fasciculus that served as a control white matter tract. “AND” ROIs were drawn on the posterior coronal slices on the separation point of temporal and frontal lobe. The first ROI was placed on the temporal lobe and the second ROI on a more caudal coronal section to include the projections toward the frontal lobe. Finally, caudal “NOT” ROI was placed to eliminate irrelevant fibers (for individual tracts, see Supplementary Figure S4). Every individual tract was visually inspected and inadequate outlier tracts were removed using additional “NOT” ROIs.

#### Hippocampal Subfields Volume

Individual volumes of the left DG (precisely referring to the left granule cell layer and molecular layer of the DG), left CA1 and left subiculum (which both served as a control hippocampal subfield) were segmented using FreeSurfer (version 6.0)^3^ algorithm, introduced by Iglesias et al. (2015). Fully automated cortical and subcortical reconstructions and volumetric segmentations, including the hippocampus were performed (for individual volumetric segmentation of all hippocampal subfields, see Supplementary Figure S5). Preprocessing of T1-weighted images included intensity normalization, skull stripping and automated topology correction using a watershed algorithm (Fischl et al., 2002). Individual left DG, left CA1 and left subiculum volumes were adjusted for intracranial volume (ICV) (den Heijer et al., 2012; Kerti et al., 2013; Kobe et al., 2017) with the following formula:

Adjusted Volume = raw volume – *b* × (ICV – mean ICV).

The coefficient *b* indicates the regression slope of the region to be adjusted on the ICV. Individual hippocampal subfields were superimposed on anatomical images. Segmentation quality was visually inspected and no further correction was necessary.

#### Statistical Analysis

**FIGURE 2:**
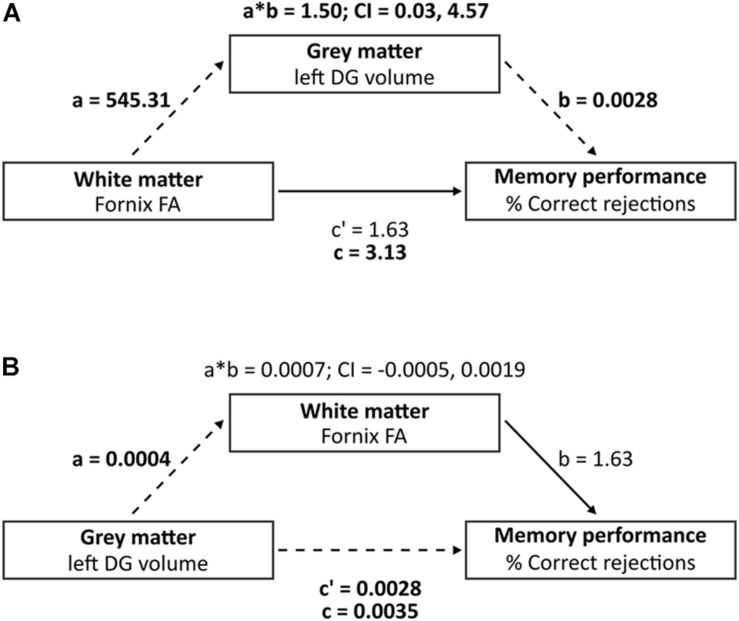
Mediation analysis investigating the three-way association between left DG volume, Fornix FA and percentage of correct rejections. **(A)** Mediation analysis (model A) where “a” indicates the effect of fornix FA on left DG volume, “b” indicates the effect of left DG volume on % correct rejections, adjusted for fornix FA, i.e., direct effect of left DG volume on % correct rejections, “c” indicates the direct effect of fornix FA on % correct rejections, and “c” indicates the total effect (direct and indirect) of fornix FA on % correct rejections. **(B)** Reverse mediation analysis (model B) where “a” indicates the effect of left DG volume on fornix FA, “b” indicates the effect of fornix FA on % correct rejections, adjusted for left DG volume, i.e., direct effect of fornix FA on % correct rejections, “c” indicates the direct effect of left DG volume on % correct rejections, and “c” indicates the total effect (direct and indirect) of left DG volume on % correct rejections. Dashed lines indicate significant paths and bold font indicates significant terms. Bias-corrected 95% CI is displayed for the indirect effects (a^∗^b).

We used SPSS 25.0^4^ to perform all statistical analyses. Partial correlation coefficients were computed for correlation analyses of brain structural variables and behavioral performance, corrected for age. As per Hartopp et al. (2018), partial correlations with hippocampal subfields, white matter microstructures and memory performance were performed. We implemented a simple mediation analysis using PROCESS (Hayes and Preacher, 2014; Zamroziewicz et al., 2017; Zhang et al., 2018) in order to test whether the relation between a predictor (fornix FA) and an outcome (percentage of correct rejections) is mediated – in total or in part – by a mediator variable (volume of left DG, and as control regions; volume of left CA1 or left subiculum) (Baron and Kenny, 1986). We assessed the indirect effect of the independent variable (IV) and the dependent variable (DV) through a mediator variable (MV), corrected for age. To test this hypothesis, a bootstrapping resampling strategy was implemented while taking 5000 bootstrap samples. Path *a* describes the direct effect of the IV (fornix FA) on the MV (left DG volume; volume of left CA1 or left subiculum), path *b* represents the direct effect of the MV (left DG volume; volume of left CA1 or left subiculum) on the DV (percentage of correct rejections), and path *c* indicates the total effect of IV and MV on the DV. Finally, path *c′* reveals the direct effect of the IV (minus MV) on the DV (Figure 2 and Supplementary Figure S6). Bias-corrected 95% confidence interval (CI) was computed to evaluate the contribution of the MV (indirect effect, path a × b). CI reached significance when the interval range did not include zero.

## Results

### Memory and Fornix White Matter Microstructure

Fornix FA showed a positive correlation with percentage of total correct responses (partial correlation coefficient: r (corrected for age) = 0.39, *p* = 0.035). In order to examine whether this relationship was specific to a response category, we further examined separate correlations between Fornix FA and percentage of hits and correct rejections: Fornix FA did not correlate with percentage of hits (partial *r* = −0.001, *p* = 0.995) but showed a positive correlation with percentage of correct rejections (partial *r* = 0.403, *p* = 0.030, Figure 1). FA values extracted from the uncinate fasciculus did not correlate with percentage of correct rejections (partial *r* = 0.299, *p* = 0.115, see Supplementary Figure S7).

### Memory and Left DG Volume

Left DG volume showed no strong correlation with percentage of total correct responses (partial correlation coefficient: *r* (corrected for age) = 0.291, *p* = 0.126). In order to explore this relationship in different response categories, we further examined separate correlations between left DG volume and percentage of hits and correct rejections: left DG volume did not correlate with percentage of hits (partial *r* = −0.133, *p* = 0.492) but showed a positive correlation with percentage of correct rejections (partial *r* = 0.501, *p* = 0.006, Figure 1). Volume left CA1 did not correlate with percentage of correct rejections (partial *r* = 0.341, *p* = 0.071, see Supplementary Figure S7). Correlations with all other hippocampal subfields were statistically non-significant (Supplementary Table S1, all *p*’s > 0.05).

### Mediation Model Analysis

Our model including fornix FA, memory performance and left DG volume met the criteria for mediation, where path a, b and c showed significant associations (*p* < 0.05) and path c′ did not show significant associations (Table 2). Single mediation analysis (model A) showed an indirect effect of fornix FA on percentage of correct rejections, mediated by left DG volume, corrected for age (β = 1.50, 95% CI: 0.03, 4.57, Table 2 and Figure 2A). The mediation effect (path ab) constituted 48% of the total effect of fornix FA on percentage of correct rejections (path c). Reverse mediation analysis (model B) was performed to investigate whether fornix FA mediates the effect of left DG volume on percentage of correct rejections, as such, we can induce the specificity of left DG volume in mediating fornix FA effect of percentage of correct rejections. Reverse mediation analysis showed that fornix FA did not mediate the effect of left DG volume on percentage of correct rejections, corrected for age (β = 0.0007, 95% CI: −0.0005, 0.0019, Table 2 and Figure 2B). These findings support the mediation effects of left DG volume on the relationship between fornix FA and percentage of correct rejections. Further mediation analysis showed that neither left CA1 nor left subiculum mediated the effect of fornix FA on the percentage of correct rejections (Supplementary Figure S6).

**TABLE 2:** Mediation analysis models of the three-way association between left DG volume, Fornix FA, and percentage of correct rejections (*n* = 30).

**Effect**	**Coefficient ± SE (% Mediation)**	***t***	***p***	**95% CI**
**Model A**				
Total effect c (fornix FA on % correct rejections)	3.13 ± 1.37	2.28	0.030*	
a (fornix FA on left DG volume)	545.31 ± 189.80	2.87	0.008**	
b (left DG volume on % correct rejections)	0.0028 ± 0.0013	2.10	0.045*	
Mediation effect ab (fornix FA on % correct rejections via left DG volume)	1.50 ± 1.03 (48)	–	–	**0.03, 4.57**
Direct effect c′ (fornix FA on % correct rejections)	1.63 ± 1.47	1.10	0.279	
**Model B**				
Total effect c (left DG volume on % correct rejections)	0.0035 ± 0.0012	3.0	0.006**	
a (left DG volume on fornix FA)	0.0004 ± 0.0001	2.87	0.008**	
b (Fornix FA on % correct rejections)	1.63 ± 1.47	1.10	0.279	
Mediation effect ab (left DG volume on % correct rejections via Fornix FA)	0.0007 ± 0.0006	–	–	−0.0005, 0.0019
Direct effect c′ (left DG volume on % correct rejections)	0.0028 ± 0.0013	2.10	0.045*	

FA, fractional anisotropy; DG, dentate gyrus* indicates significance with *p* < 0.05 and ** indicates significance with *p* < 0.01. Significant bias-corrected CI are shown in bold.

## Discussion

The current study investigated the effect of fornix white matter microstructure and left DG volume on the retrieval of episodic memory in healthy older adults. Fornix FA and left DG volume were correlated with correct rejections (i.e., successful retrieval of “incorrect” associations) during retrieval of previously acquired picture-word pairs. Mediation analysis further showed that the prediction of memory performance by increased fornix microstructure was mediated by higher volume in the left DG, but not vice versa. This finding indicates that reduced fornix microstructure impairs successful retrieval of learned associations through its impact on left DG subfield of the hippocampus.

### Association of Fornix White Matter Microstructure and Memory Performance

Our finding of a positive association between fornix FA and memory performance is in line with previous studies in both young and older adults (Rudebeck et al., 2009; Douet and Chang, 2014; Ly et al., 2016). Ly et al. (2016) found that variability in fornix microstructure in middle-to-late aged adults was related to face recognition memory and partly explained preserved functional connectivity within hippocampal networks. A study by Metzler-Baddeley et al. (2011) further suggested that specifically age-related degradation of fornix microstructure as derived from individual fiber tracking was linked to memory recall performance in strategic and visual memory tasks in older adults (Metzler-Baddeley et al., 2011). Our data confirms the role of the fornix in older adults in verbal episodic memory (Metzler-Baddeley et al., 2011, 2012). Moreover, we found a positive link between fornix FA and percentage of correct rejections in older adults, indicating that preservation of forniceal fiber pathway integrity with increasing age may be crucial for the ability to detect false associations. We found no correlation of FA of the uncinate fasciculus with percentage of correct rejections. Bennett et al. (2015) found effects of fornix FA on the prediction of pattern separation performance. They also found no effect of uncinate fasciculus and cingulum bundle FA on the prediction pattern separation performance. Taken together, these findings suggest that pattern separation performance, corresponding to the ability to detect false associations, relies selectively on fornix microstructural integrity.

### Association of Dentate Gyrus Volume and Memory Performance

In line with previous studies, age-related preservation in left DG volumes was also associated with superior memory performance (Small et al., 2002; Toner et al., 2009; Stark and Stark, 2017; Bennett et al., 2018). Our data further suggests that in particular the inhibition of false memories may be sensitive to the effects of age-related atrophy in the left DG. This result complements the findings of Shing et al. (2011) who showed that DG volumes in healthy older adults were negatively correlated to false alarm rates [which are complementary to correct rejection rates (Lynn and Barrett, 2014)] in a word-pair learning task. The authors hypothesized that the role of the DG is to enhance the specificity of encoded memories. In order to store overlapping inputs, DG performs pattern separation that enables the correct retrieval of interfering information (Rolls, 2010). As such, successful rejection of false associations is based on the efficient representation of differences between the correct and the incorrect associations. Interestingly, older adults show a specific decrease in their pattern separation ability, making them more vulnerable to memory distortions (Toner et al., 2009; Stark et al., 2010). It is also possible that this specific association between left DG volume and correct rejections reveals the function of left DG in implementing retrieval strategies to prevent these memory distortions [for review (Kesner et al., 2004)]. Novelty detection is one strategy that has been related to the DG and that allows detection of newly presented information. Our findings lend further support to this concept, assuming that participants may have implemented this strategy to successfully detect new (i.e., incorrect) associations. Additionally, we found no correlation of percentage of correct rejections with left CA1 subfield volume, confirming that this subfield may not be crucial for pattern separation (Bakker et al., 2008). This suggests that our results were specific for the left DG.

### Association of Fornix White Matter Microstructure and Memory Performance Was Mediated by Dentate Gyrus Volume

In the present study, correct rejections, used as main outcome in the mediation analysis, most likely quantified pattern separation performance that has been associated to both, DG and fornix (Shing et al., 2011; Bennett et al., 2015). Combining both white matter microstructure and gray matter volume within the structural hippocampal memory network, our mediation analysis showed that the prediction of memory performance by fornix microstructure was partially mediated by the volume of left DG in older adults. Previous studies have shown a link between age-related decrease of fornix integrity and hippocampal atrophy (Pelletier et al., 2013; Zhuang et al., 2013; Wisse et al., 2015; Gazes et al., 2018). The directional relationship, however, remains unclear, as it is conceivable that both hippocampal gray matter loss induces fornix white matter fiber degeneration and vice versa (cf. Zhuang et al., 2013). So far, it has been shown that fornix microstructural degradation, and not hippocampal atrophy, served as a biomarker for early amnestic MCI due to AD (Zhuang et al., 2013). Further, using mediation analysis, a recent study provided evidence for a causal effect of age-related fornix white matter damage on hippocampal gray matter volume decline in healthy adults (Metzler-Baddeley et al., 2019). The current study complements previous evidence by demonstrating that fornix microstructure and left DG subfield volume interact to specifically mediate memory for false associations.

It should be noted that cells of the DG do not project outside of the hippocampal formation, so this area can provide only indirect input to the fornix (Insausti and Amaral, 2004). However, Gondard et al. (2015) showed that deep brain stimulation of the fornix activated the DG by modulating the expression of neurotrophic factors and markers of synaptic plasticity known to be crucial for memory processing. This association could be explained by the interconnectivity of subfields in the hippocampus, forming a functional unit within the hippocampal formation (cf. Duvernoy et al., 2005; Wisse et al., 2015). Based on this, one can assume that structural proximity between hippocampal subfields may make it difficult to clearly distinguish individual subfield function. Rather, the specificity of hippocampal subfields function may be demonstrated by its exclusive effect on specific cognitive outcomes. In patients with MCI and AD, in particular atrophy of the subiculum was associated with fornix microstructure (Wisse et al., 2015) which may point toward different patterns of hippocampal disconnection in early AD. Future studies are needed that unveil the interconnectivity between hippocampal subfields and white matter tracts involved in this circuit.

In conclusion, our mediation model offered a neurobehavioral model in which preserved fornix white matter microstructure in older age predicts successful retrieval of learned associations through its protective effect on gray matter volume in the left DG. Thus, demyelination, even in healthy aging, may induce gray matter loss reflecting altered intracellular metabolism or neural death in connected structures, and leading to constrained memory performance (Bartzokis, 2004; Metzler-Baddeley et al., 2019). Due to the cross-sectional design of our study that still limits conclusions about causality; future longitudinal studies are needed to support this hypothesis.

### Strengths and Limitations of the Study

Strengths of the study include the multimodal imaging approach of combined gray matter volumetric analysis and white matter fiber tractography to assess the impact of structural integrity that promotes successful cognitive function in older adults. A robust tractography method was used. CSD overcomes the limitations of other DTI techniques, estimating the orientation of multiple intravoxel fiber populations in regions of white matter structures with crossing fibers like the fornix (Tournier et al., 2008; Jeurissen et al., 2011). The present study presents two methodological limitations; first, partial volume effects (PVE) affect DTI-based indices (Alexander et al., 2001; Szczepankiewicz et al., 2013). An individual voxel in brain imaging may contain different types of tissues; gray matter, white matter and cerebrospinal fluid. PVE refers to the impact that tissues, other than white matter, may have on tractography analysis, leading to underestimated FA values within a voxel [for review (Tohka, 2014)]. In order to attenuate PVE, we set the FA threshold to 0.2, as a mean to eliminate all underestimated FA values. Second, concerns about the hippocampal subfield segmentation tool implemented in FreeSurfer were recently expressed when applied on T1-weighted images with standard spatial resolution (Pluta et al., 2012; Wisse et al., 2014; de Flores et al., 2015; Yushkevich et al., 2015). Using the automated segmentation on images with 1 mm-resolution may result in less accurate delineation of boundaries within subfields. It is thus recommended to acquire T2 images to improve automated subfield segmentation (Iglesias et al., 2015). Therefore, volumetric results from hippocampal subregions must be interpreted with caution. Nevertheless, we are confident that the automated segmentation in FreeSurfer provides highly useful information, allows for reproducible and – compared to manual segmentation – less labor intensive and less biased results (Iglesias et al., 2015; Foo et al., 2017; Li et al., 2018; Zheng et al., 2018); and that individual subfields in our study were produced in the right position.

## Conclusion

We investigated the effect of fornix FA and left DG volume on retrieval of episodic memory in healthy older adults. Our findings demonstrated that preserved fornix microstructure positively impacts on memory for false associations through a protective effect on left DG subfield volume. More generally, the results lend further support to the hypothesis that structural disconnection plays a crucial role in mediating deficits in the course of aging. Our study provides a neurobehavioral model for the linkage between structural memory network properties to explain inter-individual variability behavioral outcomes in older adults. Understanding this relationship is an important prerequisite for the development of interventions to counteract cognitive decline such as aerobic exercise, cognitive training or dietary interventions (Brickman et al., 2014; Antonenko et al., 2016b; Kobe et al., 2016).

## Data Availability Statement

The datasets generated during the current study are available from the corresponding author on reasonable request.

## Ethics Statement

The studies involving human participants were reviewed and approved by the Charité – Universitätsmedizin Berlin, Corporate Member of Freie Universität Berlin, Humboldt-Universität Berlin, and Berlin Institute of Health, Department of Neurology, NeuroCure Clinical Research Center, Berlin, Germany. The patients/participants provided their written informed consent to participate in this study.

## Author Contributions

DH, DA, and AF designed the research. DH, FT, and DA analyzed the data. DH performed the tractography and volumetric analysis. DH prepared all figures. DH and DA wrote the manuscript. All authors reviewed and revised the manuscript.

## Conflict of Interest

The authors declare that the research was conducted in the absence of any commercial or financial relationships that could be construed as a potential conflict of interest.

## Footnotes

1 https://www.memoryclinic.ch/de/

2 http://www.neurobs.com/, version 18.1

3 http://surfer.nmr.mgh.harvard.edu/

4 http://www-01.ibm.com/software/uk/analytics/spss/
