# Supplementary material for: Dentate Gyrus Volume Mediates the Effect of Fornix Microstructure on Memory Formation in Older Adults
Source: Front Aging Neurosci. 2020 Mar 20;12:79. doi: 10.3389/fnagi.2020.00079 (PMC7098987; doi:10.3389/fnagi.2020.00079)
Supplement: Supplementary file 1 [file Data_Sheet_1.pdf]

Dentate gyrus volume mediates the effect of fornix microstructure on memory formation in older adults

Dayana Hayek[1,2]*, Friederike Thams [2], Agnes Flöel[1,2,3], Daria Antonenko[1,2]*

[1]Charité – Universitätsmedizin Berlin, Corporate Member of Freie Universität Berlin, Humboldt-Universität Berlin, and Berlin Institute of Health, Department of Neurology, NeuroCure Clinical Research Center, Berlin, Germany; [2]Department of Neurology, Universitätsmedizin Greifswald, Greifswald, Germany. [3]German Centre for Neurodegenerative Diseases (DZNE) Standort Greifswald, Greifswald, Germany

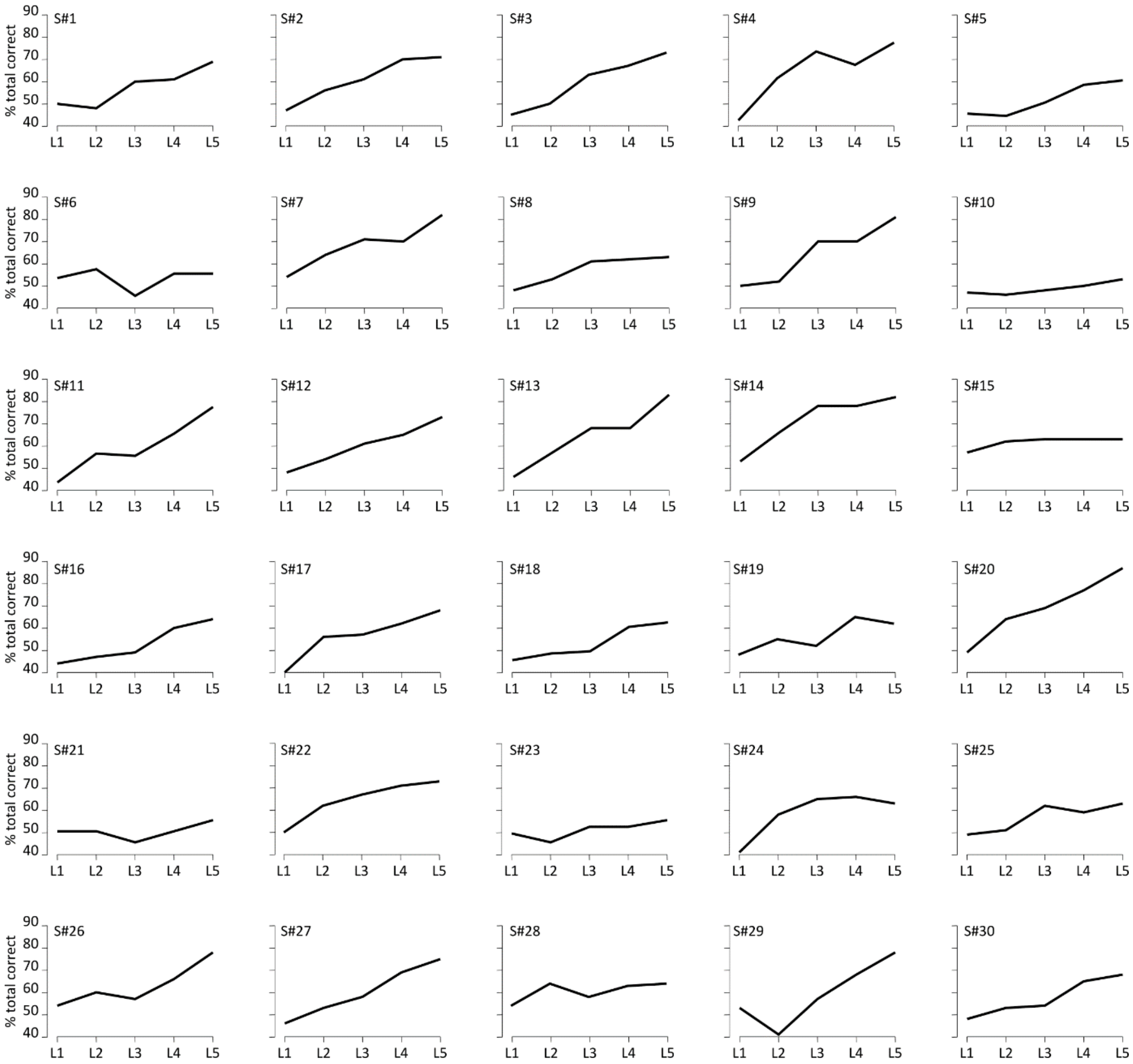

Supplementary Figure S1. Individual curves of the percentage of total correct answers in the five learning blocks (L1 to L5).

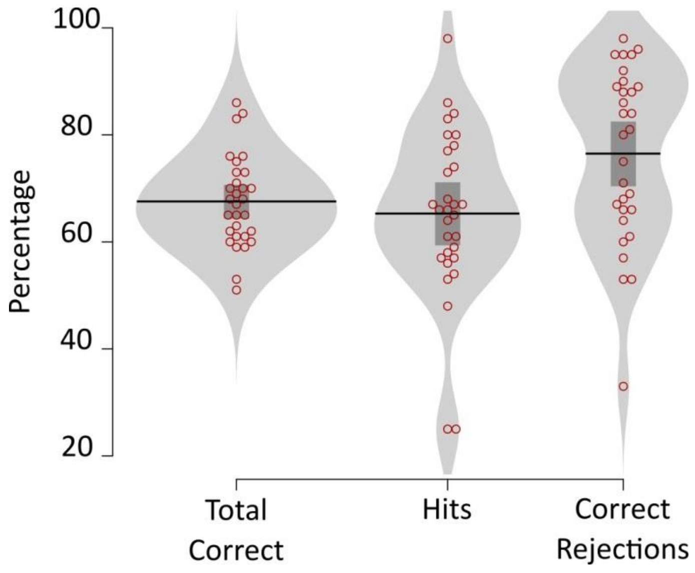

<u>Supplementary Figure S2.</u> Bean plots showing the distribution of percentages of total correct, hits, and correct rejections. Plots were constructed using BoxPlotR (Spitzer et al., 2014; http://shiny.chemgrid.org/boxplotr/). The red circles represent the individual data points, the lines represent the mean values. Error bars represent the 95% confidence intervals.

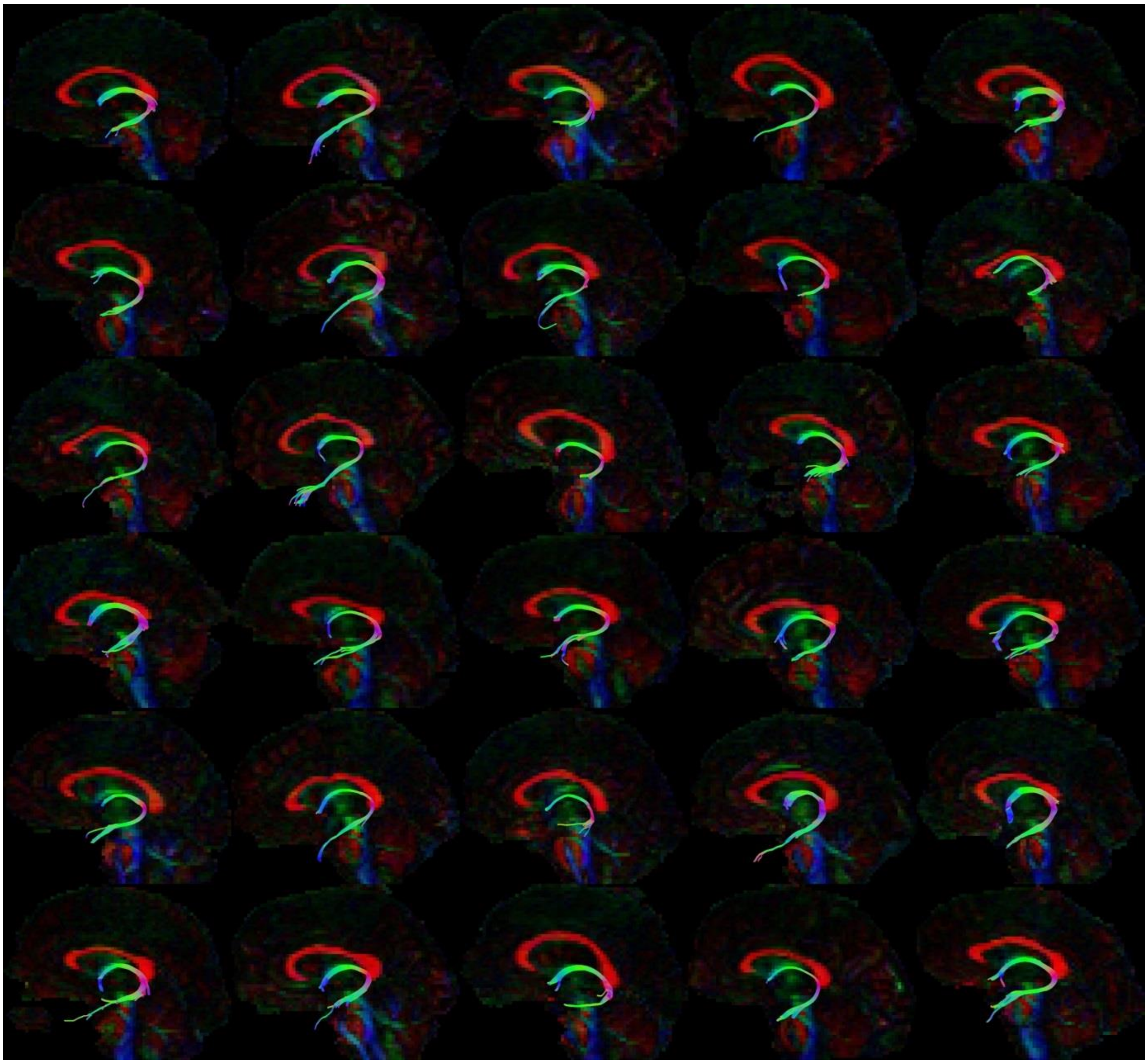

Supplementary Figure S3. Sagittal view of the left fornix in the 30 subjects. Using the ExploreDTI toolbox (Leemans, Jeurissen et al. 2009), the fornix was manually drawn by D.H. in native space using color-coded fiber orientation maps for individual subjects while blinded to age and memory performance.

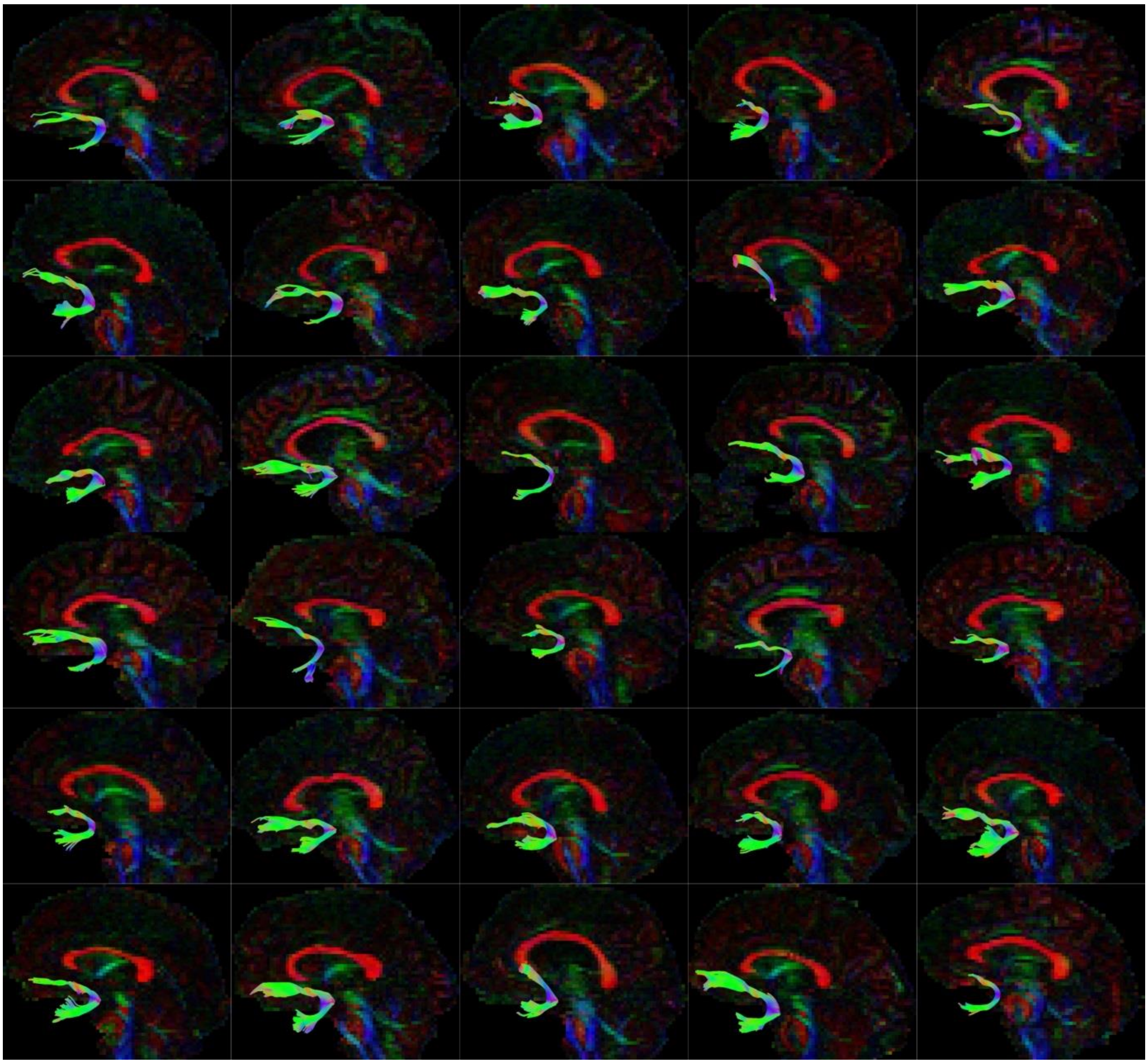

Supplementary Figure S4. Sagittal view of the left uncinated fasciculus (UF) in the 30 subjects. Using the ExploreDTI toolbox (Leemans, Jeurissen et al. 2009), the UF was manually drawn by D.H. in native space using color-coded fiber orientation maps for individual subjects while blinded to age and memory performance.

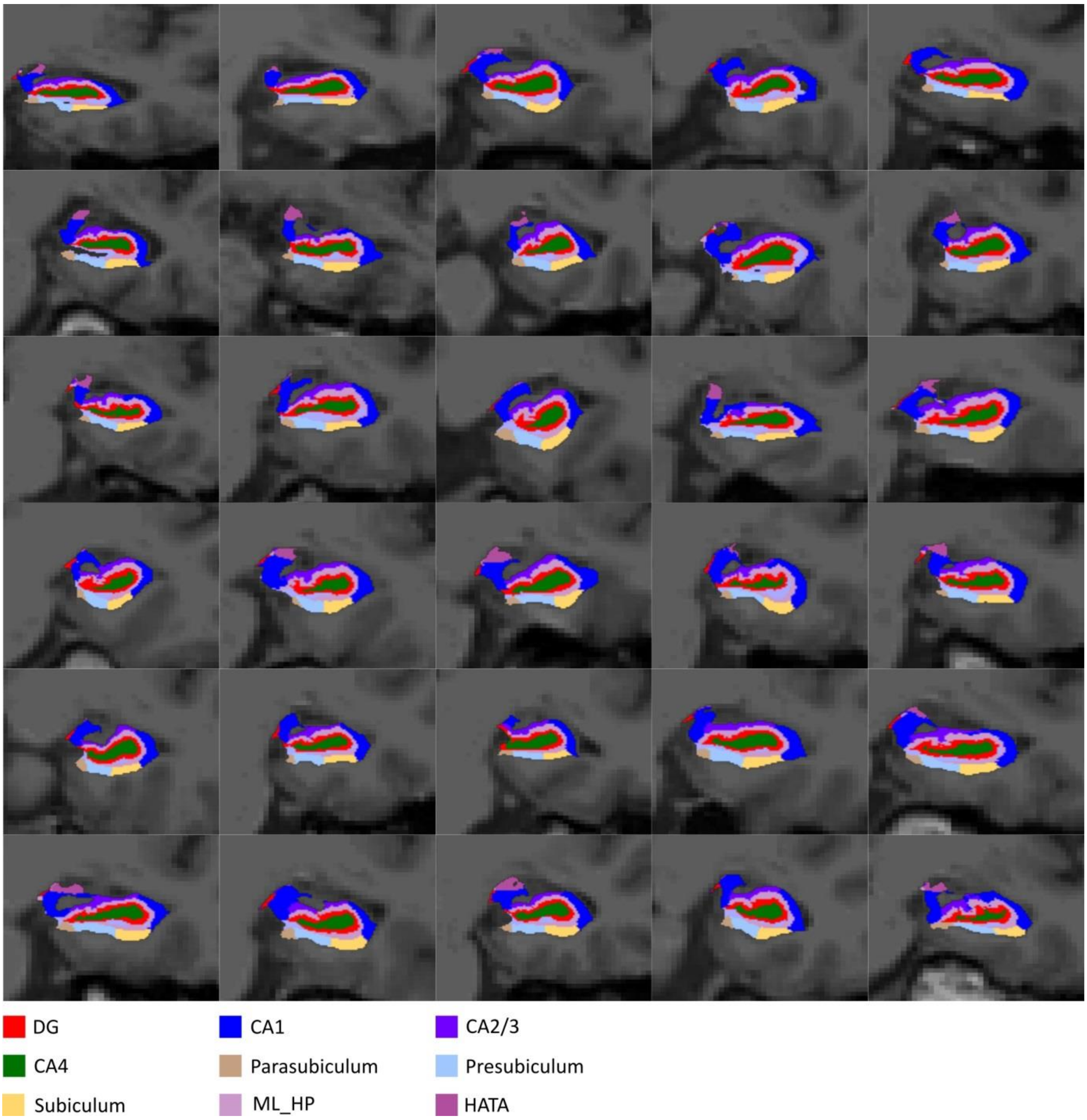

Supplementary Figure S5. Coronal view of the left hippocampal subfields in the 30 subjects. Fully automated hippocampal subfields reconstruction and volumetric segmentations were performed using the FreeSurfer (version 6.0) (http://surfer.nmr.mgh.harvard.edu/) algorithm, introduced by Iglesias et al. (2015). Legend represents all hippocampal subfields. Hippocampal fimbria, hippocampal fissure and hippocampal tail were not visible. DG: dentate gyrus; CA (1-4): cornu ammonis; ML_HP: molecular layer of the hippocampus.

Supplementary table S1. Correlation matrix presenting linear associations between hippocampal subfields volume, white matter microstructures and memory performance.

	% Corr Rej	Fornix FA	L CA1	L CA2/3	L CA4	L Sub	L DG	R CA1	R CA2/3	R CA4	R Sub	R DG
% Corr Rej	1.000											
Fornix FA	0.403*	1.000										
L CA1	0.341	0.391*	1.000									
L CA2/3	0.204	0.179	0.682**	1.000								
L CA4	0.356	0.377*	0.785**	0.739**	1.000							
L Sub	0.265	0.340	0.617**	0.270	0.697**	1.000						
L DG	**0.501****	**0.484****	0.795**	0.651**	0.941**	0.760**	1.000					
R CA1	0.130	0.207	0.813**	0.689**	0.673**	0.447*	0.637**	1.000				
R CA2/3	0.127	0.035	0.441*	0.653**	0.427*	0.030	0.397*	0.615**	1.000			
R CA4	0.269	0.264	0.686**	0.702**	0.713**	0.394*	0.700**	0.763**	0.860**	1.000		
R Sub	0.212	0.467*	0.523**	0.172	0.592**	0.786**	0.604**	.0429*	0.148	0.286	1.000	
R DG	0.338	0.274	0.680**	0.697**	0.730**	0.454**	0.747**	0.758**	0.830**	0.981**	0.302	1.000

Values represent results of partial correlation analysis with age correction. All volumes were adjusted for ICV. * indicates correlation significant at $p<0.05$ and ** indicates correlation significant at $p<0.01$. No corrections for multiple comparisons were performed. Abbreviations: % Corr Rej: percentage of correct rejections, L: left, R: right, Sub: subiculum, DG: dentate gyrus.

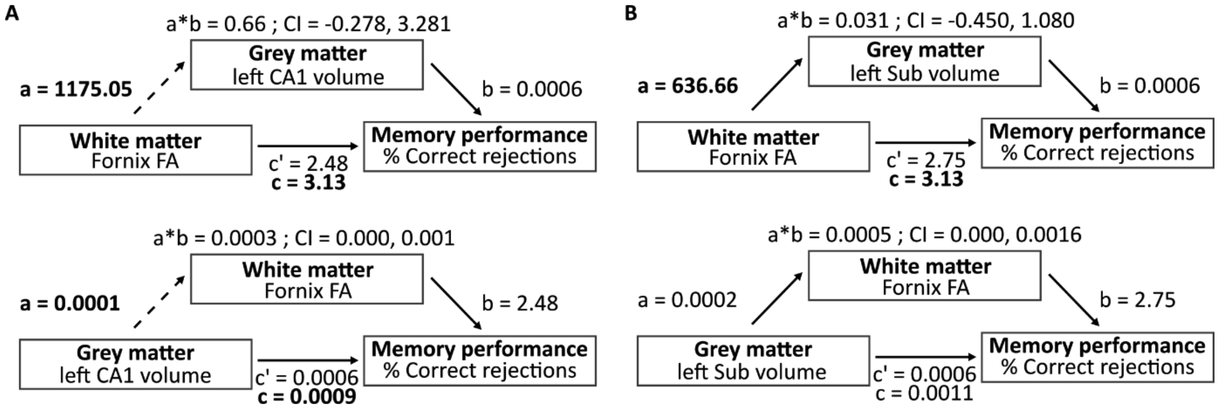

Supplementary figure S6. Mediation analysis investigating the three-way association of left CA1 volume (A) and left subiculum volume (B), with Fornix FA and percentage of correct rejections. (A) Upper panel shows mediation analysis where "a" indicates the effect of fornix FA on left CA1 volume, "b" indicates the effect of left CA1 volume on % correct rejections, adjusted for fornix FA, i.e., direct effect of left CA1 volume on % correct rejections, "c'" indicates the direct effect of fornix FA on % correct rejections, and "c" indicates the total effect (direct and indirect) of fornix FA on % correct rejections. Lower panel shows Reverse mediation analysis where "a" indicates the effect of left CA1 volume on fornix FA, "b" indicates the effect of fornix FA on % correct rejections, adjusted for left CA1 volume, i.e., direct effect of fornix FA on % correct rejections, "c'" indicates the direct effect of left CA1 volume on % correct rejections, and "c" indicates the total effect (direct and indirect) of left CA1 volume on % correct rejections. (B) Same model for left subiculum volume. Dashed lines indicate significant paths and bold font indicates significant terms. Bias-corrected 95% CI is displayed for the indirect effects (a*b) that refer to significant mediation if the CI does not include zero.

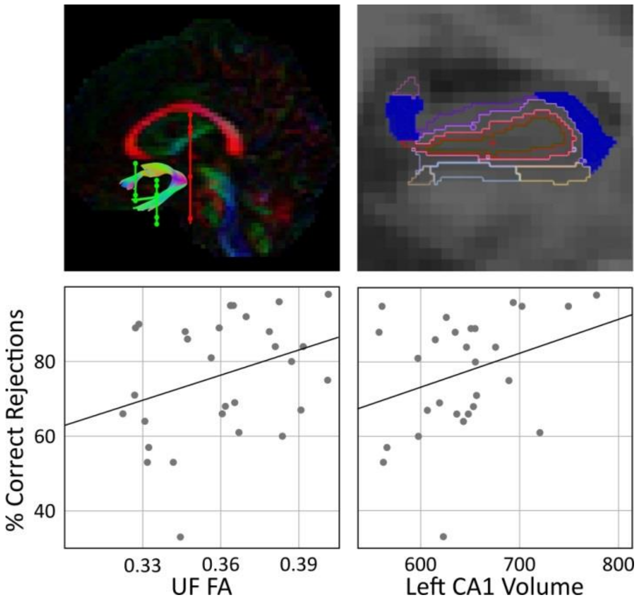

Supplementary Figure S7. Uncinate fasciculus (left panel), CA1 (right panel) and their correlation with memory performance (UF: partial r = 0.299, p = 0.115; CA1: partial r = 0.341, p = 0.071).
